# The Feeding Infants and Toddlers Study (FITS) 2016: Moving Forward

**DOI:** 10.1093/jn/nxy159

**Published:** 2018-08-31

**Authors:** Johanna T Dwyer

**Affiliations:** Tufts University School of Medicine and Frances Stern Nutrition Center, Tufts Medical Center, Boston, MA

## Introduction

The years from birth through preschool involve more changes in growth, development, eating patterns, nutrition, and other functions than any other time of life (1). Food consumption during this time is dynamic and is influenced by rapidly changing trends in feeding practices for infants and young children, as well as by longer-term trends in family incomes and food programs. It is critical to know what children are being fed, what they are eating, and how practices are changing, if we are to craft interventions that lay a solid nutritional foundation for later health, decrease risks of inappropriate eating habits, and develop evidence-based feeding recommendations.

Informal guidelines for feeding the young have been available since antiquity. Today, we use formal evaluation of the evidence before making recommendations (2–5). Of particular relevance here is the Birth to 24 Months (B-24) project to evaluate data and support the addition of the first ever recommendations for children younger than 24 mo to the 2020–2025 Dietary Guidelines for Americans (6). Sound recommendations must be based on up-to-date information, and yet data on intakes and eating patterns are sparse, particularly for those <24 mo of age.

NHANES provides much useful information for children <24 mo of age, but the sample sizes of both breast- and bottle-fed infants and toddlers are insufficient to trace the rapid changes in intakes that occur during that time (7, 8). The Feeding Infants and Toddlers Study (FITS) 2016 contributes to this evidence base and complements NHANES by applying similar methods to a large sample of infants and toddlers aged <24 mo, including minorities, providing greater detail about the adequacy of usual nutrient intakes and the foods and food groups consumed.

FITS 2016 is a cross-sectional study of caregivers of children under the age of 4 y living in the 50 states and Washington DC. Data collection occurred between June 2015 and May 2016. A recruitment interview (respondent and child characteristics, feeding practices including responsive feeding and reasons for starting or stopping breastfeeding, physical activity, screen use, sleep habits, participation in food assistance programs) was completed by telephone or online. This was followed by a feeding practices questionnaire and a 24-h recall conducted by telephone. A second 24-h recall was collected for a random subsample of 25% of the total sampled population. Because FITS is a telephone survey, direct anthropometric data could not be collected; the lack of accurate anthropometric data or other biomarkers to link the food consumption data collected to health outcomes is a limitation (9).

## Key Findings

### Infants: breastfeeding, breast milk substitutes, and introduction of complementary foods

Roess et al. (10) found that the initiation and duration of breastfeeding appear to be increasing in the total population over the 3 FITS surveys (11), and with FITS 2016, the percentage of mothers initiating breastfeeding and the percentage continuing to breastfeed at 12 mo both met goals set by Healthy People 2020 (12). However, the prevalence and duration of exclusive breastfeeding still fell short of the Healthy People 2020 goals. Guthrie et al. (13) found that infants participating in WIC were less likely to be put to the breast at all, and if they were, were breastfed for a shorter time than non-WIC participants.

Breast milk substitutes were mostly appropriate (i.e., iron-fortified infant formulas), and older infants typically transitioned from formula to cow milk around 12 mo, in accordance with pediatric feeding guidelines that recommend delaying the introduction of cow milk until 12 mo (14, 15). The prevalence of early introduction of cow milk has stayed roughly the same since 2008: use of cow milk rather than formula in early and middle infancy (0–8.9 mo of age) was rare (10).

Complementary feeding is an area of active research and discussion (16). The prevalence of introducing solids <4 mo of age continued to decline (10), compared with FITS 2008 (11), confirming NHANES data that also suggest a lower prevalence of complementary feeding in very young infants than previously reported (8). However, infants consuming any formula were more likely to be introduced to complementary foods <4 mo of age (10), whereas, paradoxically, infants participating in WIC (who are more likely to be fed formula) were less likely to be introduced to complementary foods <4 mo of age (13). This suggests that WIC recipients are influenced by what foods WIC provides (i.e., for very young infants, WIC provides formula, but not complementary foods).

Among older infants, fewer consumed iron-fortified baby cereal and iron-fortified formula (10) than reported in FITS 2008 (17). At the same time, few infants were fed iron-rich puréed baby meats. These patterns raise concerns about the adequacy of iron intakes for some older infants that are borne out by Bailey et al.’s (18) finding that a greater percentage of older infants consumed less than the estimated average requirement of iron in 2016 than in 2008, putting them at increased risk of iron deficiency. Bailey et al. (18) do not break out nutrient intakes by food source, so these iron results cannot be definitively linked to consumption rates for these foods at this time, but they are consistent and suggest avenues for future investigation. In contrast, Jun et al. (19) found that WIC participants were less likely to be at risk of iron deficiency than nonparticipants.

Fruits and vegetables were being introduced to infants at an appropriate age, and fruit juice consumption was slightly lower than prior levels and reflected amounts closer to recommendations. In contrast, the early introduction of inappropriate complementary foods, including fruit-flavored drinks and presweetened cereals, remains a concern (10).

The appropriateness of energy intakes compared with body composition could not be measured accurately in a phone survey, so this aspect of intake could not be assessed. Except for iron, as noted above, nutrient intakes were adequate for most infants (18). The use of dietary supplements for younger infants has increased substantially since 2008, largely attributable to the increased use of vitamin D supplements in keeping with American Academy of Pediatrics (AAP) recommendations for the use of vitamin D supplements in infants. However, some solely breastfed infants may not be getting all the vitamin D they need as they approach later infancy (18), in part because the acceptable intake was increased from 5 (20) to 10 µg/d in 2011 (21), and feeding practices may not have changed in line with that.

### Toddlers and young children: transition to family diets

For toddlers (ages 12–23.9 mo), transitioning from breastfeeding or infant formula and appropriate complementary foods to family diets continues to be somewhat problematic (22). Young children (ages 24–47.9 mo) do eat healthy foods, but often only small quantities, whereas they eat much larger amounts of high-calorie items. Intakes of undesirable nutrients such as sodium and saturated fat often exceeded adequate intakes or acceptable macronutrient distribution ranges, whereas intakes of more desirable nutrients such as fiber, vitamin D, and potassium too often fell short of recommendations (18). Also, energy intakes often need attention.

#### Breast milk and cow milk

A higher proportion of toddlers (ages 12–17.9 mo) consumed breast milk on the day of the survey than in past FITS studies (10). Most toddlers and young children drink cow milk, although there is room for increased consumption of low-fat milk among young children. Toddlers were most likely to drink whole milk as recommended. Welker et al. (23) found that young children (ages 24–47.9 mo) were about equally likely to drink whole milk, 2% milk, and 1% milk, but few drank skim milk, highlighting a need to continue efforts to shift from whole to lower-fat varieties at age 24 mo. WIC children were more likely to make this shift to lower-fat varieties at ages 24 mo than were non-WIC children (13).

#### Whole grains

More than half of toddlers and young children consumed whole grains or whole grain–rich foods (>50% whole grain), but these were mostly in cereals, and to a lesser extent breads, rolls, bagels, and tortillas. Very few consumed whole-grain crackers, pasta, or rice, so there is room for improvement there.

#### Vegetables

Although most toddlers and young children consumed some vegetables, too many did not consume a distinct vegetable portion on the day of the recall. Among those who did, there was a clear lack of variety, depriving children of experience with different textures, colors, sound, tastes, and flavors (10). Recent NHANES surveys confirm these findings (8, 24). White potatoes remained the most commonly consumed vegetable, as in 2008, although children were eating fewer fried potatoes. However, unlike 2008, when WIC infants were much less likely to be consuming vegetables than non-WIC infants, WIC infants in 2016 were about equally likely to be consuming vegetables as non-WIC participants were, and are generally more likely to consume baby-food vegetables and less likely to consume nonbaby-food vegetables than nonparticipants are (13). Thus, the addition of baby-food vegetables to the WIC package may have been helpful in bringing about this improvement.

#### Fruit and fruit juice

Positive changes in consumption patterns since 2008 include continued declines in fruit juice consumption and increased consumption of fruits, which have lower caloric density than the juices. However, some children consumed no fruit on the day of the survey. Despite declines in fruit juice consumption, juice was still introduced too often before 12 mo, and amounts for toddlers (ages 12–23.9 mo) were often above the new AAP recommendation to limit juice to 4 oz (118 mL) per day (10). WIC children were considerably more likely to consume juice than were non-WIC participants were (13).

#### Proteins

Young children consumed enough protein, but often not from the healthiest sources, with more consumption of protein foods high in sodium and saturated fats, and less of healthier choices such as lean meats, seafood, nuts, seeds, soy, and legumes (23). This is consistent with the nutrient analysis findings in Bailey et al. (18) that show young children too often consume too much sodium and saturated fats.

#### Sweets, sugary beverages, and savory snacks

Although intakes of sugar-sweetened beverages, desserts, sweets, and salty snacks were down slightly compared with FITS 2008, the prevalence of consumption remained high, and amounts eaten often exceeded targets for discretionary calorie consumption (23). Thus, consumption of these foods increased calories, sugars, and salt, often at the expense of more nutrient-dense foods. In addition to these concerns, frequent consumption of foods high in sugar or starch increases the risk of dental caries (25). Similar trends were also evident in NHANES (26). WIC children aged 24–47.9 mo were less likely to consume sugar-sweetened beverages than were lower-income (and likely WIC-eligible) non-WIC participants (13). In neither survey could energy intakes vis-à-vis body composition be assessed accurately.

### Intakes of WIC participants compared with non-WIC participants

The WIC program serves lower-income pregnant, breastfeeding, and postpartum women, and infants and young children. The program reaches 51% of infants and 28% of all US children <5 y of age. Therefore, the eating behaviors of WIC participants compared with nonparticipants are of great interest.

Initiation, duration, and exclusivity of breastfeeding for WIC infants all fell short of the same measures for non-WIC infants. Mothers of young WIC infants (0–5.9 mo of age) were most likely to use formula and not breastfeed at all, or only partially breastfeed. Exclusive breastfeeding among this population was less common than among non-WIC infants. Less than a third of mothers of older WIC infants (6–11.9 mo of age) breastfed at all, and very few did so exclusively. Therefore, breastfeeding promotion remains an important area for further nutrition education. However, mean nutrient intakes were higher for WIC infants. Compared with both lower- and higher-income non-WIC infants, WIC infants had higher intakes of iron, zinc, and vitamin D, which more often fell short in non-WIC infants. This may be attributable to the higher use of formula, which is usually fortified.

For toddlers (12–23.9 mo old) and young children (24–47.9 mo old), the WIC nutritional advantage was smaller than for infants, and intakes were closer to those of non-WIC children. WIC children were more likely to shift to lower-fat milk options at age 2 y, consistent with expert recommendations, but they were also considerably more likely to consume fruit juice than non-WIC children were (13). Usual nutrient intakes of WIC toddlers and young children were adequate, and WIC children had higher intakes of iron and lower risk of iron deficiency at all ages (19), but there is room for improvement in other respects: WIC children at all ages had higher sodium intakes than non-WIC participants, and their vitamin A (retinol) and zinc intakes exceeded the tolerable upper limit more often.

Implementing the changes in WIC food packages recommended by the 2017 National Academies of Sciences, Engineering, and Medicine (NASEM) report (27), to increase whole grains, decrease juice, and increase the value of vouchers that recipients can use to purchase fruits and vegetables, should help in correcting some of these problems.

## Needs for Future Research

Much remains to be learned about the nutrient requirements of the very young, what and how they are fed, and strategies to foster healthful food consumption habits among them. Needs for future research include the following.

### Better data on the food patterns of infants

NHANES is particularly well positioned to provide better data on feeding patterns that will be needed for evidence-based guidelines on feeding infants, but it needs and must receive additional resources to do so (28). These resources must be made available with funding from interested federal agencies or Congressional appropriations to permit NHANES to collect larger samples and to oversample poor and minority infants and children, especially those <2 y of age, with samples large enough to analyze differences in feeding practices by race/ethnicity, income, and age. Samples also need to be augmented for pregnant and lactating women.

Although FITS 2016 provides solid new information on food patterns, accurate measures of weight and height/length for age could not be obtained in telephone interviews, making it impossible to link food patterns to weight, growth, and health-related behaviors. NHANES, on the other hand, includes both intakes and the anthropometric, biochemical, and clinical measurements that are vital to provide a complete picture of the nutritional status of young infants and the potential impacts of dietary patterns on child development, weight, linear growth, and health and food preferences. However, neither survey provides accurate data on the volume of breast milk consumed by infants. In addition, the timing of foods introduced may influence food allergies (29). Future research should be expanded to study these factors.

### Better data on nutrient requirements of infants

With the exceptions of iron and zinc for infants >6 mo of age, who have established estimated average requirements, RDAs, and tolerable upper limits, data are so limited from experimental studies that nearly all nutrient requirements <1 y of age are extrapolated from adult data and are adequate intakes, not RDAs. More experimental studies are needed, but these will be difficult for young infants, because quantitative estimates require test weighing to estimate volume of breast milk consumed (which was not done here, owing to it being a phone survey). The DRIs, particularly for vitamins A and E, zinc, fiber, and potassium, need review and possible revision.

### Better methods to improve accuracy of breast milk intake

Better methods are needed to assess the intakes of breastfeeding infants (30). Breast milk nutrient composition values in databases also need updating and must reflect how the nutrient composition of breast milk changes as the infant grows. Very little is known about the variability in nutrient composition of human breast milk. More information on partial and mixed breastfeeding patterns is also needed to assess their nutritional and health effects.

Dietary supplements are now an important part of total nutrient intakes of many US children (31–34). At least a quarter of FITS 2016 children took dietary supplements containing nutrients (18). NHANES needs to continue to document the influence of dietary supplements on child intakes and health. There is also a need for a complete database on the nutrient composition of supplements reported by caretakers. Some supplements, such as vitamin D and iron for breastfed infants in later infancy, are appropriate and fill nutrient intake gaps, whereas others provide nutrients in amounts that may cause total intakes to exceed the tolerable upper limit. Of concern in FITS 2016 were excessive intakes of retinol, zinc, and, to a lesser extent, folic acid among older children who used supplements containing those nutrients. Formulations of vitamin-mineral supplements for children <5 y of age need to be reviewed and better tailored to reflect child requirements (35). The rationale for, and effects on nutrient adequacy and excess of, multivitamin/mineral supplements for young children who are users and nonusers should also be studied (36, 37).

### Effects of multiple caretakers and child care settings on intakes

Most US children <5 y of age are placed in nonparental child care facilities such as child care centers, preschools, Head Start, or homecare outside their families by the time they are preschool-age. Most of the food and beverages consumed away from their homes in these facilities are difficult for parents to quantify (38). It is important to know more about the effects of different caretakers and care settings on dietary patterns and nutrient intakes, food selection, weight status, food enjoyment, food safety risks, and health.

### Vegetable and fruit consumption

Better understanding of the conditions under which children eat or reject vegetables and fruits is needed to develop more effective interventions to encourage children to eat them (39). The attitudes toward and consumption of vegetables and fruits by parents, caretakers, and other family members also deserve study.

### Effects of federal food programs on intakes, weight, and health

The prevalence of excess weight for length/height (>95th percentile) is ∼10% for infants and children aged <2 y, 8.9% for children aged 2–5 y, and 17–21% for children aged ≥6 y (40–43). Among WIC participants from 3 to 23 mo of age, the prevalence of high weight for length from 2000 to 2014 ranged from a high of 14.5% in 2004 down to 12.3% in 2014 (44). FITS 2016 shows that the WIC program is associated with enhanced intakes of certain nutrients for infants and children in families relying on it to supplement their diets (19). It is important to keep WIC and other safety-net programs strong; at the same time, it is vital to evaluate the effects of those programs and any revisions in them (e.g., changes to the WIC food package recommended by a NASEM Committee in 2017) (27).

### Evidence-based guidelines for feeding infants and toddlers

In developing evidence-based guidelines, nutrition and healthcare professionals have an opportunity to guide child-feeding norms in more healthful directions. There is a pressing need for nutrition education to ensure that infants’ and young children's intakes are developmentally appropriate, enjoyable, and nutritionally adequate without being excessive. From FITS 2016, it appears that stronger recommendations are needed so that parents and caretakers understand the following:

- that feeding is most successful when it is done in a manner that responds to the child's hunger and satiety cues (45);
- the specific foods children should be eating and the developmentally appropriate times to introduce complementary foods and beverages; and
- the appropriate frequency and portion sizes of discretionary high-calorie, high-fat, high-sugar, and high-salt foods and snacks.

Parents and other caretakers must know what others are feeding the child to ensure children are properly fed. Parents can also help by being good role models, serving and eating healthy meals, and keeping healthier food choices (such as a variety of vegetables, fruits, and lean protein and low-fat dairy foods) on hand so that they are easily accessible, and making high-calorie sugary/sweet foods and savory snacks less available.

## FITS moving forward

The papers in this supplement provide only a glimpse at the findings of FITS 2016, and preliminary, informal comparisons with FITS 2002 and 2008. FITS 2016 is moving forward, with more analyses planned on food sources of nutrient intakes; formal trend analysis from 2002 to 2016 for intakes; and correlations between intakes and sleep, physical activity, and screen use. A critical evaluation of infant feeding practices and responsive feeding will also be forthcoming. More detailed analyses of Federal program participation and how that influences dietary patterns are also ongoing. Similar studies are also now underway in other countries, including China (46–48), Mexico (49, 50), and Russia (51), and will soon be underway in the Philippines, India, the Middle East, and Brazil. These will continue to inform us about the similarities and differences in infant and child feeding practices between countries (52).

## Conclusions

The good news is that infants <1 y of age generally have adequate nutrient intakes, and preliminary comparisons suggest that, in some ways, feeding patterns have improved since 2008. At all ages, children had satisfactory intakes of B vitamins, vitamins C and K, and most minerals except potassium. However, nutrients such as fiber, vitamins D and E, potassium, and (in some infants) iron began to deviate downward, whereas both sodium and consumption of high-calorie, high-sugar foods and beverages began to escalate upward from recommendations in toddlers, becoming even more pronounced among young children. The family eating patterns that young children transition to need improvement. Imbalances between calorie-dense but not nutrient-dense foods and more nutrient-dense foods (such as whole grains, fruits, and lean protein foods) are apparent in family diets. Both parents and their young children need to be discriminating if they are to improve their intakes. They must learn to eat more like gourmets than gourmands. This entails eating foods in appropriate amounts; being selective without being picky; and exploring a variety of different tastes, textures, colors, and flavors. In crafting guidance to help parents and caretakers modify inappropriate feeding patterns, FITS 2016 provides much that will be helpful.

## Notes

Published in a supplement to *The Journal of Nutrition*. Select contents of this supplement were presented at the Experimental Biology 2017 conference in a session titled, “Informing B-24 Dietary Guidelines: Findings from the New Feeding Infants and Toddlers Study 2016,” held in Chicago, IL, April 22, 2017. Supplement Coordinators were William Dietz, Milken School of Public Health at the George Washington University and Andrea S. Anater, RTI International. The FITS research was funded by Nestlé Research (Lausanne, Switzerland) through a contract with RTI International and its subcontractor, the University of Minnesota. Dr. Dietz is a compensated consultant to RTI on this project. The article contents are the responsibility of the authors and do not necessarily represent the opinions or recommendations of Nestlé. Publication costs for this supplement were defrayed in part by the payment of page charges. This publication must therefore be hereby marked “advertisement” in accordance with 18 USC section 1734 solely to indicate this fact. The opinions expressed in this publication are those of the authors and are not attributable to the sponsors or the publisher, Editor, or Editorial Board of *The Journal of Nutrition*.

Supported by Nestlé Research, Lausanne, Switzerland.

Author disclosures: JTD is a member of the Scientific Advisory Boards of Conagra Foods and McCormick Spice, has served as a consultant for Gerber/Nestlé, and has received partial travel expenses to moderate a symposium at the International Congress of Nutrition in Buenos Aires, Argentina in 2017 from the International Alliance of Dietary Supplement Associations. She holds stock in several food and drug companies.
